# Amyand’s Hernia: Incarcerated Appendicitis in a Recurrent Inguinal Hernia in an Adult

**DOI:** 10.7759/cureus.53528

**Published:** 2024-02-03

**Authors:** Jignesh B Rathod, Haryax V Pathak, Kartik P Ajediya, Ravi K Bhatt

**Affiliations:** 1 Surgery, Pramukhswami Medical College, Shree Krishna Hospital, Bhaikaka University, Karamsad, IND; 2 Surgery, Surgery, Pramukhswami Medical College, Shree Krishna Hospital, Bhaikaka University, Karamsad, IND; 3 General Surgery, Surgery, Pramukhswami Medical College, Shree Krishna Hospital, Bhaikaka University, Karamsad, IND

**Keywords:** advanced and complicated hernia surgeries, incarcerated inguinal hernia, appendicitis, recurrent inguinal hernia, amyand’s hernia

## Abstract

Inguinal hernias are among the most common cases presented to a surgeon. In spite of extensive research and clinical experience over centuries, inguinal hernias still pose anatomical challenges for operating surgeons, especially with a propensity for recurrence. One such complicated entity is the Amyand's hernia - defined as an inguinal hernia contained within the hernial sac - the vermiform appendix - as the herniated content. It is a rare clinical presentation and carries with it certain complexities with regard to operative decisions and clinical management. We present a case of a 71-year-old male presenting with a recurrent inguinal hernia, with an incarcerated, inflamed appendix as the content; managed surgically with appendicectomy and herniorraphy, without the use of a prosthetic mesh.

## Introduction

Hernia is defined as the protrusion of a viscus or a part of the viscus through a normal or abnormal opening in the wall of the cavity that normally contains it. Usually, the content of hernial sacs is the omentum or the bowel. Hernias vary in type and clinical presentation, like umbilical, inguinal, femoral, incisional, epigastric, etc. Inguinal hernias are one of the most common cases encountered by a surgeon, with nearly 1.5 to two million inguinal hernia surgeries being conducted annually in India [1]. They are widely described in the literature with regard to types, clinical findings, and surgical techniques for management. Despite extensive research, inguinal hernias, even today, have the propensity to present with unusual and rare findings, often requiring complex surgical maneuvers for management.

Amyand’s Hernia is defined as an inguinal hernia contained within the hernial sac - the vermiform appendix - as the herniated content. It was first described in the literature by Claudius Amyand in 1735 when he discovered a ruptured appendix as the content of a congenital inguinal hernia in an 11-year-old male [2]. It was managed surgically by resection of the appendix as well as the hernial sac. Since then, sporadic cases of Amyand’s hernia have been reported in the literature, with the overall incidence being a mere 1% of all inguinal hernias [3,4]. Another similar entity is the de Garengeot hernia, wherein the vermiform appendix forms the content of a femoral hernia [3,4].

Amyand’s hernia is usually encountered in the neonatal or pediatric age groups, likely because of a patent processus vaginalis. The age of presentation though has a wide range, with reported cases in a three-week-old patient as well as a 92-year-old patient [5,6]. As per a study by Sharma et al, of all cases of Amyand’s hernia, the incidence of acute appendicitis is extremely rare - barely 0.07%-0.13% [7]. Pre-operative diagnosis is rare, with ultrasonography suggesting herniation of bowel contents through the defect. Contrast-enhanced computed tomography (CECT) of the abdomen and pelvis may demonstrate herniation of the appendix - but a CECT is rarely done for diagnosis of an inguinal hernia. Acute appendicitis in an Amyand’s hernia may further progress to perforation and abdominal sepsis, associated with a mortality rate of 15%-30% [8]. We present a case of a 71-year-old male presenting with a recurrent inguinal hernia, with an incarcerated, inflamed appendix as the content without peritonitis; managed surgically with appendicectomy and herniorraphy.

## Case presentation

A 71-year-old male, with no known co-morbidities presented with a complaint of swelling in the right groin region for two months, appearing on straining and coughing, but spontaneously disappearing upon lying down. Gradually, the swelling became persistent for one week, approximately 3x3 cm in size, associated with complaints of pain over the swelling for three days. The patient did not have any addictions like smoking or alcoholism. The patient had a history of left-sided open inguinal hernioplasty 25 years ago, and right-sided open inguinal hernioplasty 12 years ago.

On presentation, the patient was vitally and hemodynamically stable, with a pulse rate of 88 beats per minute, blood pressure of 126/78 mmHg, and respiratory rate of 16/min, maintaining saturation of 99% on room air. The patient was afebrile, with no pallor, icterus, cyanosis, edema, or lymphadenopathy. The patient had scars from the previous surgeries in bilateral inguinal regions, 5 cm in length, running obliquely along and 1.25 cm above the line joining the anterior superior iliac spine and pubic tubercle, consistent with history of previous bilateral open inguinal hernioplasty.

Upon examination, the patient had a swelling in the right inguinal region, which was 3x3 cm in size, pyriform shaped, irreducible, tender, with a soft consistency, reaching just above the scrotum, originating at the mid-inguinal point, overlying the scar of previous surgery. A clinical diagnosis of a right-sided irreducible inguinal hernia was made. Since the patient had a history of previous hernioplasty on the right side, it was concluded that the patient was having a recurrent right inguinal hernia.

Ultrasonography of the abdomen and pelvis suggested a 10-mm defect in the right inguinal region, with herniation of omentum and bowel loops. Routine hematological and biochemical investigations were suggestive of leucocytosis with an elevated neutrophil count.

In view of past surgical history and recurrence of inguinal hernia, it was decided to post the patient for laparoscopic hernia repair and do totally extraperitoneal hernioplasty (TEP), as TEP provides faster recovery times, lesser wound complications, and better pain management over open inguinal hernia repair, especially in cases of recurrent hernias wherein the abdominal wall muscles are already weak and have reduced integrity [9].

**Figure 1:**
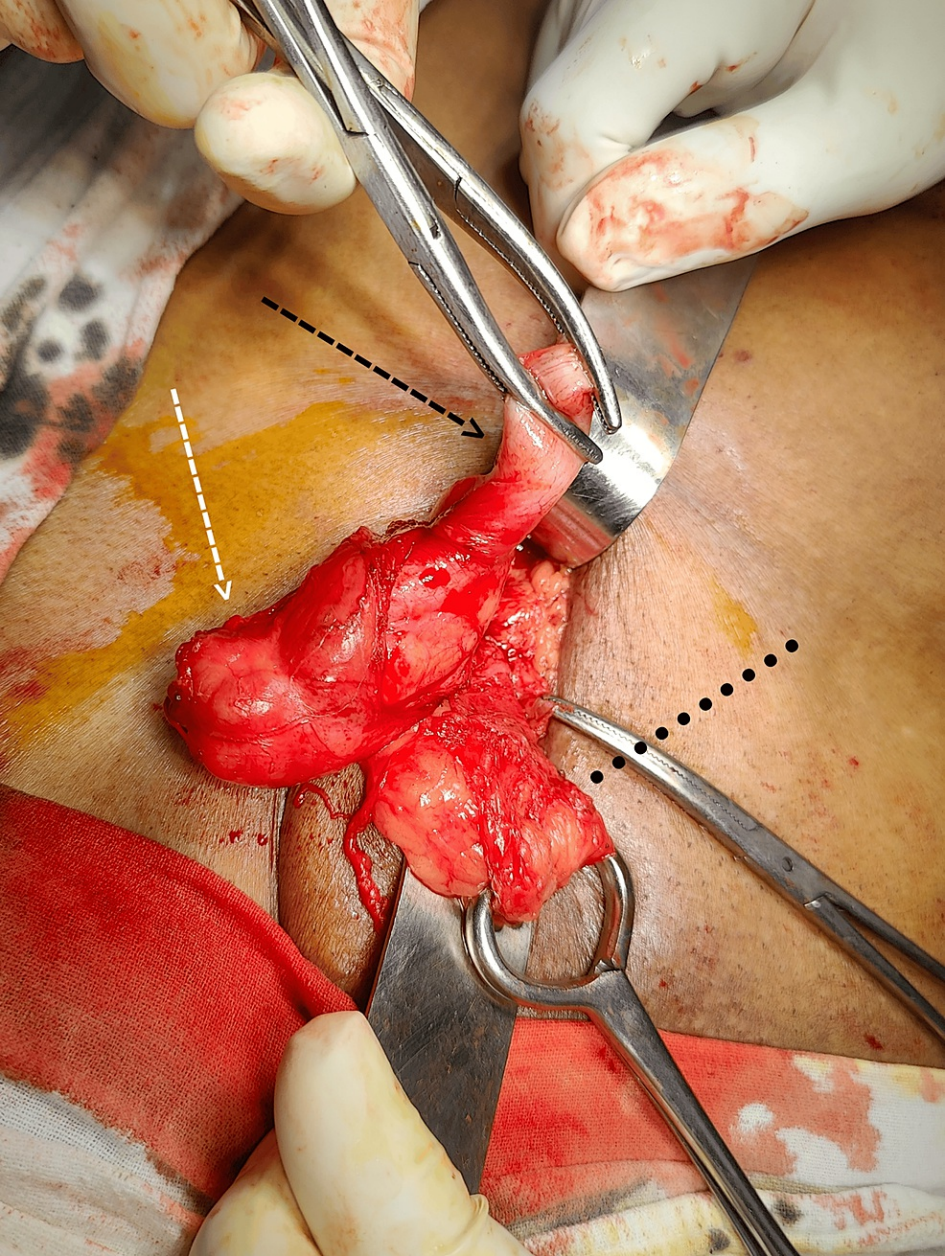
Contents of the hernial sac Black Arrow: Appendix, White Arrow: Caecum, Dotted Line: Spermatic Cord

Intra-operatively, a singular blind-ending bowel loop was found to be incarcerated in the hernial sac, with tight adhesions. Multiple attempts were made to reduce the contents but failed - necessitating the conversion of laparoscopic to open hernia repair. A 5-cm oblique incision was kept in the right inguinal region, just over the scar of previous surgery. The mesh was found to be completely fibrosed with dense adhesions with surrounding tissues and thick fibrotic bands. Upon open dissection of the hernial sac, there were signs of inflammation. The appendix and a part of the caecum were found to be herniating through the defect, and thus an intra-operative diagnosis of Amyand’s hernia was made. The spermatic cord was isolated and secured. The appendix exhibited signs of active inflammation (Figure 1).

**Figure 2:**
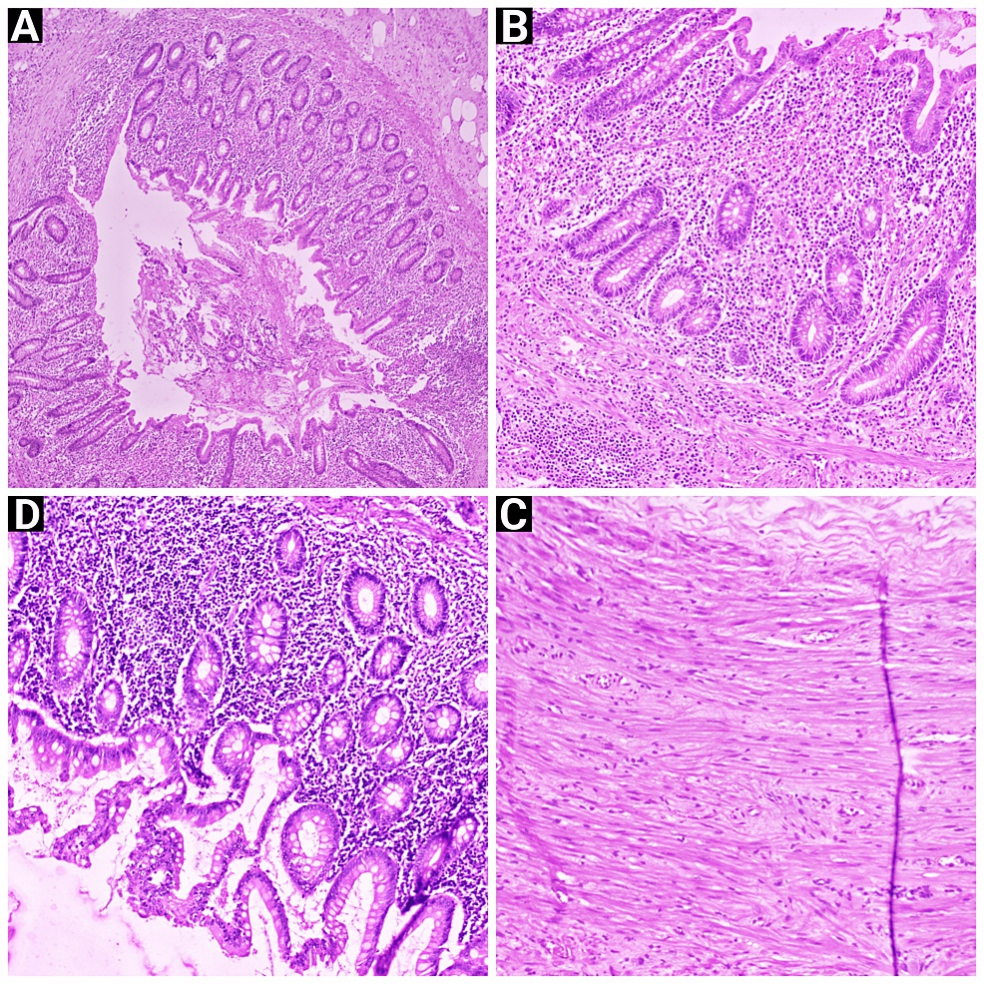
Histopathological examination of the specimen Tiles A, B, and C show 10 x 10 magnification images of the Hematoxylin - Eosin stained specimen. The micrograph shows appendiceal architecture with ulcerated and partially sloughed mucosal lining with inflammatory infiltrates and submucosal gland proliferation, as well as neutrophilic infiltrates in the muscularis layer. (A) 10x10 image showing sloughed-out mucosa of the appendix with glandular proliferation (B) 10x10 image showing pre-dominantly neutrophilic infiltrates with submucosal glandular proliferation (C) 10x10 image showing muscularis propria with neutrophilic infiltrates (D) 40 x 10 magnification image of the hematoxylin-eosin-stained specimen. The micrograph shows moderately mixed inflammatory infiltrates, comprised predominantly of polymorphs (neutrophils) and few lymphocytes, plasma cells, and occasional eosinophils. The above histopathological findings are consistent with a diagnosis of chronic appendicitis.

In view of appendicitis, an open appendicectomy was done and the appendix was sent for histopathology. As there was surrounding infection and inflammation, the hernial sac was excised and the defect was primarily closed, without placement of a prosthetic polypropylene mesh, to avoid infection of the mesh and ensuing wound infection. Histopathological examination of the specimen was suggestive of chronic appendicitis with predominantly neutrophilic infiltrates and few lymphocytes (Figures 2A-2D).

The patient recovered uneventfully and was discharged on postoperative day 2. Subsequent follow-ups one week later and one month later were unremarkable.

## Discussion

Amyand’s hernia - defined as the herniation of the vermiform appendix in an inguinal hernia - is a rare surgical entity, comprising barely 1% of all inguinal hernias, with acute appendicitis reported in 0.07% to 0.13% of cases [6]. It was first described in 1735 by Claudius Amyand, a French-born English surgeon, who conducted the first reported appendicectomy when he encountered an appendix as the content of the hernial sac while operating upon an 11-year-old male [2]. Before this, Rene Jacques Croissant de Garengeot described in 1731, the de Garengeot hernia - appendix as the content in a femoral hernia [3,4].

Amyand’s hernia can be seen in both children and adults - although it is much more common in the neonatal and pediatric age groups, because of a patent processus vaginalis. It presents as a tender irreducible swelling in the right inguinal region, but without the classical signs of acute appendicitis. It may be later complicated by the development of acute appendicitis.

Although the exact mechanism for progression to acute appendicitis has not yet been established, a review from Patoulias et al. enlists several theories for the same - [8] a) The appendix may become incarcerated and subsequently develop inflammation. b) Development of adhesion between the appendix, the serosal membrane, and the hernial sac may lead to irreducibility and predispose the appendix to infection. c) The herniated appendix may become compressed and obstructed due to the activity of the muscles of the anterior abdominal wall, further increasing the risk of inflammation and infection. d) An already inflamed appendix leads to venous stasis and initiates a vicious cycle of increasing edema and venous stasis, impairing the appendiceal microcirculation, bacterial growth, and infection.

Acute appendicitis in Amyand’s hernia may be further complicated by perforation of the appendix, leading to widespread abdominal wall sepsis and peritoneal sepsis, culminating in a mortality rate of 15%-30% [8]. After Claudius Amyand’s first description of Amyand’s hernia, several case reports have been published, describing the clinical presentation and surgical management techniques for the same. Losanoff and Basson published a detailed review of Amyand’s hernia, classified the hernia on the basis of the status of the appendix, and defined the surgical management for each type (Table 1) [10].

**Table 1:** Losanoff and Basson's classification of Amyand's hernia

TYPE OF HERNIA	SALIENT FEATURES	SURGICAL MANAGEMENT
Type 1	Normal appendix in the inguinal hernial sac	Reduction of hernia or appendicectomy (depending on age) with prosthetic mesh hernioplasty
Type 2	Acute appendicitis localized in the sac with no abdominal sepsis	Open Appendicectomy through hernia, primary repair of hernial defect without prosthetic mesh
Type 3	Acute appendicitis with abdominal wall sepsis / peritonitis	Laparotomy and Open Appendicectomy, primary repair of hernial defect without prosthetic mesh
Type 4	Acute appendicitis with concomitant abdominal pathology	Same as Type 3 with management of abdominal pathology

Singal et al. in 2012 reported three cases of incarcerated acute appendicitis in an inguinal hernia, one of them occurring in an incisional hernia in the inguinal region. They included incisional hernias to Losanoff and Basson’s classification of Amyand’s hernia, termed as Rikki’s modification, and described the management for the same (Table 2) [11].

**Table 2:** Rikki’s modification of Losanoff and Basson’s classification of Amyand’s hernia Rikki's modification includes the management of Amyand's hernia in an incisional hernia

TYPE OF HERNIA	SALIENT FEATURES	SURGICAL MANAGEMENT
Type 1	Normal appendix in the inguinal hernial sac	Reduction of hernia or appendicectomy (depending on age) with prosthetic mesh hernioplasty
Type 2	Acute appendicitis localized in the sac with no abdominal sepsis	Open Appendicectomy through hernia, primary repair of hernial defect without prosthetic mesh
Type 3	Acute appendicitis with abdominal wall sepsis / peritonitis	Laparotomy and Open Appendicectomy, primary repair of hernial defect without prosthetic mesh
Type 4	Acute appendicitis with concomitant abdominal pathology	Same as Type 3 with management of abdominal pathology
Type 5A	Normal appendix in the incisional hernial sac	Reduction of hernia or appendicectomy (depending on age) with prosthetic mesh hernioplasty
Type 5B	Acute appendicitis within incisional hernia sac with no abdominal wall sepsis / peritonitis	Open Appendicectomy through hernia, primary repair of hernial defect without prosthetic mesh
Type 5C	Acute appendicitis with abdominal wall sepsis / peritonitis or pathology related to previous surgery	Same as Type 4

We report here a case of a right-sided recurrent inguinal hernia in a 71-year-old male with the appendix as a content - Type 5B as per Rikki’s modification of Losanoff and Basson’s classification of Amyand’s hernia, managed with open appendicectomy and primary repair of the hernia without the use of a prosthetic mesh.

Prosthetic mesh use in contaminated wounds is contraindicated due to the risk of wound infection. At the same time, recurrent inguinal hernias mandate the use of a prosthetic mesh to prevent recurrence. In stark contrast to the general recommendations, Velimezis et al. report a similar case of a recurrent inguinal hernia with incarcerated acute appendicitis managed with open appendicectomy and hernioplasty with placement of an e-polytetrafluoroethylene (e-PTFE) patch [12]. Another case report by Ranganathan et al. also demonstrates the use of a prolene mesh in recurrent inguinal hernia with acute appendicitis as the content [13]. Velimezis et al. also recommend the use of an acellular dermal matrix as a safer alternative to prosthetic mesh in such situations to reduce the risk of wound infections, considering that mesh placement in recurrent inguinal hernia is a mandatory requirement [12,14]. Chatzimavroudis et al. also report the use of a polypropylene mesh in the repair of an inflamed Amyand’s hernia, accompanied by thorough drainage and toileting of the inguinal wound and antibiotic therapy [15]. Based on their study, broad-spectrum antibiotic therapy for three to five days following the surgery can prevent mesh and wound infection, and the use of a prosthetic mesh is recommended in the absence of a perforated appendix. In our case, the patient was managed without the use of a mesh. Two months following the surgery, the patient reports no complaints.

To the best of our knowledge, there have only been five previously reported cases of acute appendicitis in a recurrent inguinal hernia [12,13,15-17]. Further studies are required to make stronger recommendations backed by the literature.

## Conclusions

Amyand’s hernia remains an extremely rare condition, especially when complicated by appendicitis. As pre-operative diagnosis is difficult, the surgeon must display great clinical acumen to diagnose it intra-operatively and make surgical decisions based on the clinical findings as well as the radiological and sonological findings. The management remains surgical, with open appendicectomy and repair of the hernial defect - although the use of a prosthetic mesh with appendicitis remains a highly debatable topic. Comprehensive studies are required to examine the benefits as well as the demerits of using a mesh in an inflamed Amyand’s hernia, especially in recurrent inguinal hernias.
